# Prevalence, risk factors and management of common mental health disorders in Cameroon: a systematic review

**DOI:** 10.1136/bmjph-2023-000224

**Published:** 2024-05-30

**Authors:** Che Henry Ngwa, Limkile Mpofu, Tchokokam Patricia, John Njuma Libwea, Rejoice Uche Obiora, Marion Keinamma, Danga Aloleko Ines, Ngo Valery Ngo, Egbe Henrieta Oneke, Crayton E Bessong, Ngozi Margaret Oguguah, Emmanuel Kah, Frankline Sevidzem Wirsiy, Eman Sobh

**Affiliations:** 1 Department of Epidemiology and Population Health, American University of Beirut, Beirut, Lebanon; 2 Health Economics, HIV/AIDS, Research Division (HEARD), University of KwaZulu Natal, Durban, South Africa; 3 District Medical Center Obili, Yaounde, Cameroon; 4 Directorate for Disease Control, Epidemics and Pandemics, Ministry of Public Health, La Habana, Cuba; 5 Tampere University, Tampere, Finland; 6 Department of Community Health and Health Promotion, American University of Beirut, Beirut, Lebanon; 7 Pan Africa Christian University, Nairobi, Kenya; 8 National School of Agro-Industrial Sciences (ENSAI), University of Ngaoundere, Ngaounde, Cameroon; 9 Denis and Lenora Foretia Foundation, Yaoundé, Cameroon; 10 Department of Nursing-A&H Institute of Health Management, Limbe, Cameroon, Limbe, Cameroon; 11 Department of Physical Geography and Ecosystem Science, Lund University, Lund, Sweden; 12 Fisheries Resources, Nigerian Institute for Oceanography and Marine Research Lagos Nigeria, Lagos, Nigeria; 13 Africa Capacity Building Foundation, Harare, Zimbabwe; 14 Africa Centres for Disease Control and Prevention, Addis Ababa, Ethiopia; 15 Cameroon Baptist Convention Health Board (CBCHB), Yaoundé, Cameroon; 16 University of Nebraska Medical Center, Omaha, Nebraska, USA; 17 Chest Diseases Department, Faculty of Medicine for Girls, Al-Azhar University, Cairo, Egypt; 18 Respiratory Therapy Department, College of Medical Rehabilitation Sciences, Taibah University, Madinah, Saudi Arabia

**Keywords:** Epidemiology, Community Health, Mental Health, Prevalence

## Abstract

**Objectives:**

This review aimed to synthesise evidence on (1) the prevalence of common mental health disorders (MHDs) in Cameroon; (2) the effectiveness of mental health management interventions; (3) the risk factors for common MHDs in Cameroon and (4) map the state of research on common mental disorders in Cameroon.

**Design:**

A systematic review.

**Data sources:**

We performed a comprehensive search for articles in major public health databases including PubMed, Scopus, Medline, CINAHL, PsycINFO, Web of Science, Embase, CINAHL from inception of each database to June 2022.

**Eligibility criteria:**

Published articles on the prevalence, risk factors or intervention for management of common MHDs in Cameroon met the inclusion criteria for this systematic review.

**Data extraction and synthesis:**

The abstract and full-text screening, and data extraction were performed independently by at least two researchers. The results have been reported based on a narrative synthesis.

**Results:**

After the screening stages, 32 articles met the inclusion criteria and were included in this study. A high prevalence of common MHDs among different groups, including teenage mothers, students and people living with HIV, was observed. We identified important risk factors for these conditions in the general population and among high-risk groups. We also identified two interventions which show promising results for the management of depression among individuals with HIV in Cameroon.

**Conclusion:**

Our review recorded a high prevalence of common MHDs and identified important risk factors for MHDs among different groups. Increased priority and participatory action with all stakeholders including individuals, communities/policy holders and in research where a huge gap remains to be filled, is crucial in reducing the burden of MHDs in Cameroon.

**PROSPERO registration number:**

CRD42022348427.

**WHAT IS ALREADY KNOWN ON THIS TOPIC**

- Mental health disorders (MHDs) account for a huge loss in health quantified as disability-adjusted life-years and productivity.

**WHAT THIS STUDY ADDS**

- This study provides the first synthesis of evidence on MHDs in Cameroon and reports estimates of the prevalence of different MHDs in different populations and the associated risk factors for each MHD.
- This study highlights a huge gap in research on MHDs in Cameroon.
- This review reports the findings of two interventions with promising results for managing depression among people living with HIV.

**HOW THIS STUDY MIGHT AFFECT RESEARCH, PRACTICE OR POLICY**

- This study highlights the need for increased efforts towards MHDs across all sectors in Cameroon, including the areas of research in healthcare through improved access and quality of care and at the policy level through the development and careful monitoring of the implementation of strategies to reduce the burden of MHDs in Cameroon.

## Introduction

 Mental health disorders (MHDs) are a major global health concern, affecting people of all ages and races.^1^ These conditions have significantly increased during the last decade, with one in eight persons worldwide estimated to be living with some form of MHD in 2019.^2^ The last 3 years have particularly seen a drastic increase in the prevalence of MHDs exacerbated by the COVID-19 pandemic, with initial estimates revealing a 26% and 28% increase in anxiety and major depressive disorder, respectively.^2^ Several factors have been implicated in this increase in the prevalence of MHDs, including stress caused by COVID-19 restrictions and lockdown, fear of infection, interruption of healthcare services and inability to seek care, working conditions, loneliness and grief after bereavement.^3^ Considering the increasing burden of MHDs, careful monitoring and reporting are crucial to developing adequate prevention and sustainable care programmes, especially in resource-low settings.

In most low-income and middle-income countries, the prevalence of common MHDs is among the highest in the world.^4^ This high prevalence has been attributed in part to the difficult living conditions in the majority of these countries,^4^ which is exacerbated by fragile health systems and governments, frequent conflicts affecting many of these countries and lack of political will coupled with inadequate presence and access to care for MHDs. Common MHDs here refer to the most frequently reported MHDs in primary care^5^ or the most prevalent in the population.^2^ These include depression, anxiety, post-traumatic stress disorder (PTSD), schizophrenia, substance use and mood disorders, as reported by the WHO.^2^ These MHDs account for a considerable loss in health—quantified in disability-adjusted life-years (DALYs), a metric that takes into consideration the years of life lost due to early death and years of life lived with disability.^1^ Additionally, huge losses in productivity have been attributed to MHDs.^6^ However, due to a lack of effort and priority in research and policy, these conditions have taken the place of a ‘neglected non-communicable’ disease in most sub-Saharan African (SSA) countries.

Cameroon, an LMIC, has a similar situation to the rest of SSA, with a high prevalence of MHDs. In 2017, MHDs in Cameroon accounted for over 2360 DALYs per 100 000 populations.^7^ Also, the global burden of disease reported that about 704, 874 and 48 thousand people in Cameroon were living with major depressive disorder, anxiety disorder and schizophrenia, respectively, in 2019.^1^ These numbers are expected to have increased in the last few years, particularly in Cameroon’s English regions, partly due to COVID-19, the ongoing armed conflict, and a lack of adequate and accessible mental healthcare services.

With the introduction of Cameroon’s first mental health plan in 2016, growing interest is shed in the area of mental health research seen by the increasing number of studies.^8^ Constant monitoring and synthesis of evidence from such research will provide reliable estimates of the prevalence of common MHDs, identify high-risk groups and guide the development of policy and the implementation of mental health programmes in Cameroon. Also, the results will be helpful in guiding the implementation of the WHO Mental Health Gap Action Programme, which aims at scaling up services for mental, neurological and substance use disorders in Cameroon.^9^

This systematic review is the first on MHDs in Cameroon and aimed to synthesise evidence on (1) the prevalence of common MHDs in Cameroon; (2) the risk factors driving these conditions within the Cameroonian population; (3) the effectiveness of available interventions for managing mental health conditions in Cameroon and (4) to map the state of research on common MHDs in Cameroon.

## Methods

This review has been reported following the Preferred Reporting Items for Systematic Reviews and Meta-analyses (PRISMA) guidelines.^10^ The protocol for this systematic review was registered in the international prospective register of systematic reviews (PROSPERO registration number: CRD42022348427).

### Study inclusion criteria

Our review was limited to peer-reviewed articles published in both English and French in which (1) the data reported were collected in Cameroon; (2) the study reported the prevalence, risk factors or management of common MHDs as defined by this review and (3) the study design includes cross-sectional, case–control or cohort and randomised or non-randomised studies of interventions.

### Exclusion criteria

Review articles and case reports were excluded from this systematic review. Additionally, studies focusing on other mental health conditions not defined as common MHDs, according to our review, were excluded. Non-peer-reviewed studies, including preprints and organisational reports, were also excluded.

### Search strategy

We developed a search strategy consisting of both MeSH terms and keywords. We systematically searched electronic databases from the inception of each database to June 2022 for peer-reviewed articles, including PubMed, Scopus, Medline, PsycINFO, Web of Science, Embase, CINAHL, African Index Medicus, WHO Global Health Library, Cochrane Library, Cochrane Central Register of Controlled Trials (CENTRAL), ClinicalTrials.gov and the WHO international clinical trials registry platform. The search strategy was developed in Medline (online supplemental table 1) and applied to other databases. The search was performed in an identical fashion across all databases using the advanced search option that allows for searching the population and outcome terms together. In databases without this option, we searched for each MHD separately and limited our search to Cameroon. We also screened the bibliography of all included studies to identify potentially eligible studies that may not have been identified through the database search.

### Study selection and data extraction

The title and abstract for each article were screened independently by two researchers.

**Figure 1:**
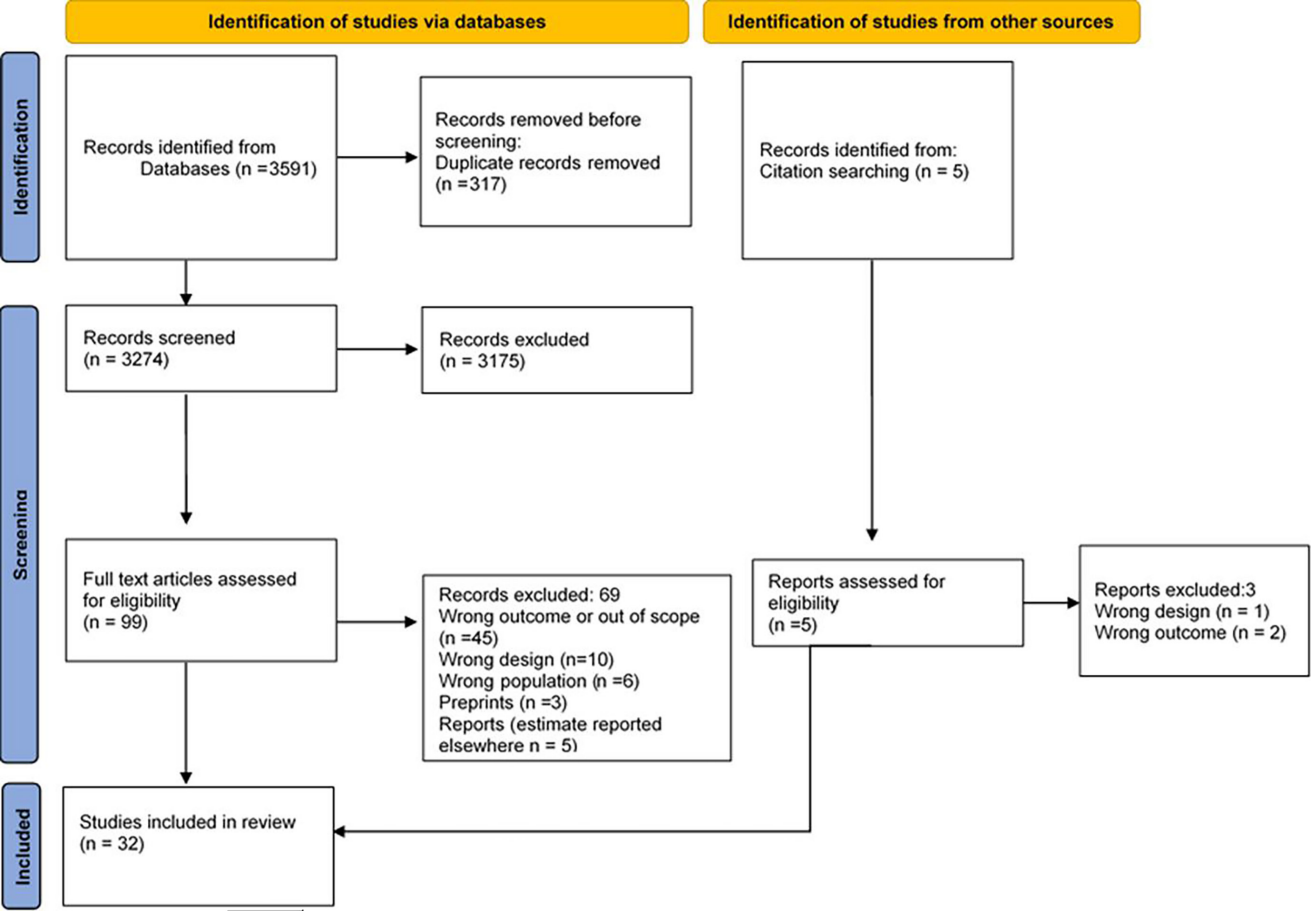
Prisma flow diagram.

All included articles in the title and abstract screening phase were assessed for eligibility independently by two researchers during the full-text screening stage. A summary of the number of articles included at each stage has been presented using a PRISMA flow diagram^11^ (figure 1). Furthermore, to ensure high-quality standards and transparency of the study outcomes, each screening phase began with a training exercise and thereafter, a pilot test with 15% of the records was performed to ensure that every team member fully understood the inclusion criteria and could use the screening guide and data extraction forms without any difficulties. This was followed by screening the full records when at least a 90% agreement rate between reviewers was achieved. A 90% agreement during the pilot was set a priori as a means to ensure that everyone understood the training material otherwise, a follow-up training session was being conducted.

### Data extraction

Data extraction for included studies was performed independently by two researchers using a standard data extraction form developed in Excel. Data extracted for each study included general information such as author name, year of publication, language and study location. We also collected information on the research method for each study, including study participants, sample size, the age range of participants, data collection method and choice of data collection instrument. Additional information on the number of persons who screened positive for each MHD and the choice of scale or instrument was collected to answer the objective on prevalence. To identify the risk factors for each MHD, we extracted information on significant risk factors from the adjusted model in each article (multivariable regression) for each MHD outcome, including the effect size and associated CI.

To report on the effectiveness of interventions for MHDs, we included a description of the design of the intervention, the population and extracted information on the preintervention and postintervention results. Any disagreements between the two researchers were resolved through a discussion between the researchers, followed by a decision from a third reviewer if the discussion proved unsuccessful.

Using data on the location of data collection for each article and publicly available data on administrative boundaries, we generated a map illustrating the state of research on common mental health conditions in Cameroon, highlighting areas where no research has been conducted.

### Risk of bias

We assessed the included studies for risk of bias using the Newcastle-Ottawa scale for assessing the risk of bias for case–control and cohort studies. We used an adapted version of the Newcastle-Ottawa scale for cross-sectional studies to assess the risk of bias for cross-sectional studies in this review. Additionally, we employed the ROBINS-I tool to assess the risk of bias for non-randomised intervention studies.

### Data synthesis

As a result of significant heterogeneity in the conduct and reporting of results of the included studies, a narrative synthesis was used to present the results.

## Results

The database search yielded 3591 records, and after eliminating duplicates, the titles and abstracts of 3274 studies were screened. A total of 99 articles from the database search were screened and assessed for eligibility at the full-text stage. 32 articles met the inclusion criteria for this review, of which 30 were obtained from the database searches and 2 from screening the bibliography of the included articles (figure 1).

### Characteristics of included studies

Out of the 32 included studies, the majority were conducted among people living with HIV,^12^,^24^ followed by students,^25^,^30^ hospital patients (including patients with diabetes, cancer and tuberculosis),^31^,^34^ pregnant and postpartum women,^15 35 36^ general population,^37^ female sex workers^38^ and children^38 39^ (online supplemental table 2)

**Figure 2:**
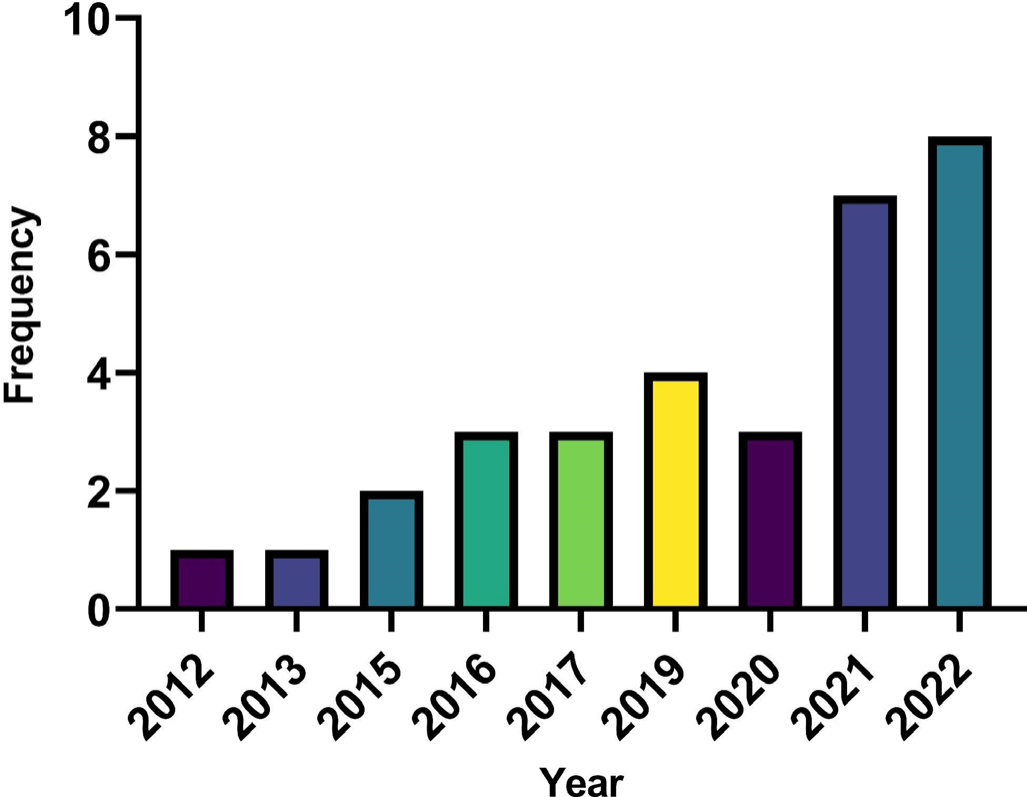
Year of publication of all included studies.

The year of publication of the different studies ranged from 2012 to 2022, with the majority of studies published in 2021 and 2022 (figure 2).

**Figure 3:**
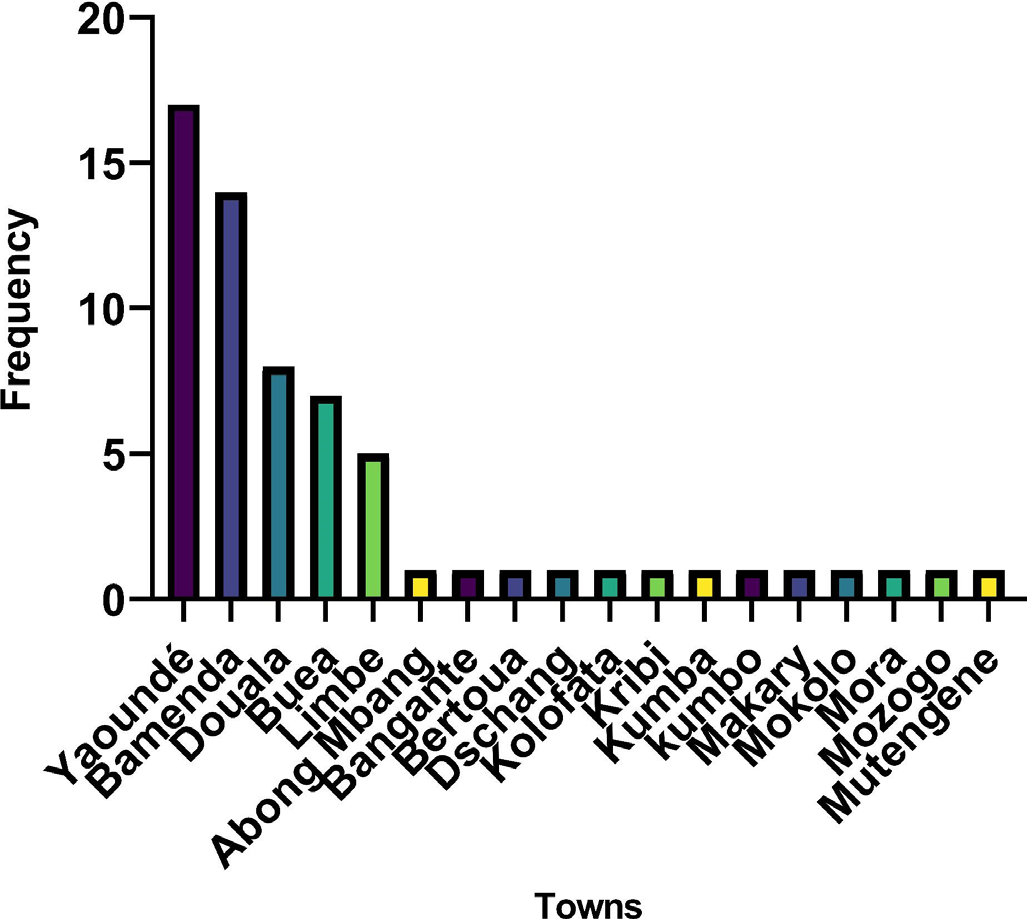
Location of data collection for all included studies.

The majority of data collection for the included studies was performed in Yaoundé, Bamenda and Douala. Also, it is worth mentioning that a good proportion of these studies involved data collection from multiple sites (figure 3).

**Figure 4:**
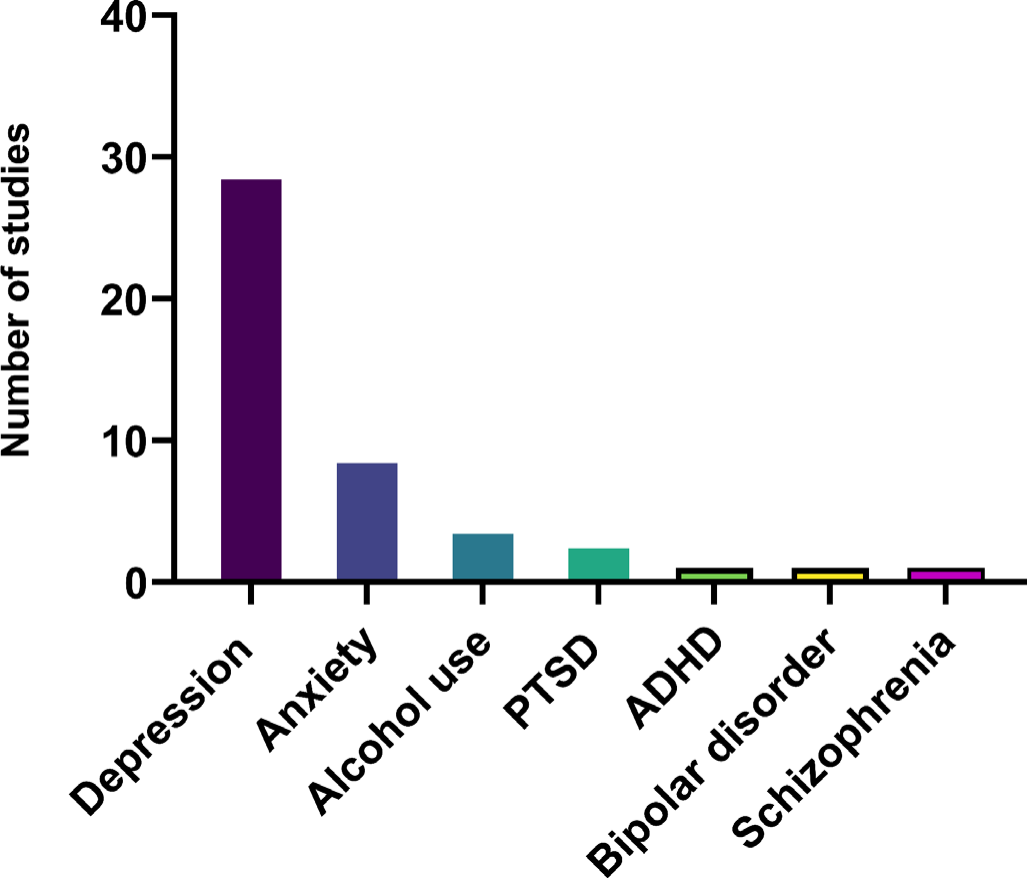
Mental health conditions from included studies.

Depression was the highest mental health condition examined by the included studies, followed by anxiety (figure 4), with a good number of studies examining multiple mental health conditions. Online supplemental figure 1 displays the location of data collection of the primary studies on a map highlighting locations where little or no research has been performed.

### Prevalence of common MHDs

The prevalence of common MHDs varied significantly between studies, the population studied and the measurement instrument employed. Online supplemental table 3 provides details on the prevalence of MHDs recorded in each study.

### Depression

The prevalence of depressive disorder in the general population ranged from 8.4%^37^ measured with Patient Health Questionnaire (PHQ-9) to 18.5% among female community inhabitants measured using the Mini-International Neuropsychiatric^40^ Interview.

In the same light, the prevalence of depression among people living with HIV ranged from 3.4% among newly diagnosed persons with HIV measured using PHQ-9^21^ to 63% among people actively on antiretroviral therapy (ART) measured with PHQ-9.^20^ Despite a few studies reporting a low prevalence, the majority of studies reporting the prevalence of depression among people living with HIV reported values ≥20%.^12 20 22^

A depression prevalence of 70% was recorded among teenage mothers using the Edinburgh postnatal depression scale^35^ while 47% was recorded among cancer patients using the Hospital Anxiety and Depression Scale (HADS).^32^

The prevalence of depression among medical students ranged from 12%^27^ using specialist-confirmed diagnoses to 30.6% using the PHQ-9.^29 30^ Also, a prevalence of 31% was recorded among nurses using PHQ-9.^41^

### Anxiety

The prevalence of anxiety in the general population was 10.8% for males and 13.1% for females using the Mini-International Neuropsychiatric Interview.^40^ Anxiety prevalence among people living with HIV was approximately 12% using the General Anxiety Disorder-7 scale,^12^ 50% for patients with cancer with HADS,^32^ 81.4% among COVID-19 patients measured with HADS,^33^ 24.7% among children and adolescents measured with Diagnostic and Statistical Manual of Mental disorder-5 (DSM-5)^39^ and 31% among medical students confirmed by a physician.^27^

### Prevalence of other common MHDs

Two studies reported the prevalence of PTSD ranging from 15% among people living with HIV^12^ to 17% among children and adolescents^39^ using DSM-5. The prevalence of mood disorders was 8.2% among children and adolescents.^39^ A prevalence of 10.5%–13.4% was reported for alcohol use disorder.^14 40^ Additionally, the prevalence of schizophrenia ranged from 7.9% to 10.1%, 8.6% to 13.6% for bipolar disorders^40^ and 24.4% for ADHD among medical students.^27^

### Risk factors

Several risk factors were identified from the different studies for the different MHDs and population groups. Online supplemental table 4 summarises the risk factors that were found to be significant with multivariable regression analyses based on findings from the different individual eligible studies.

### Depression

Several risk factors, including age,^21 33^ income and employment status,^16 21 36^ the presence of chronic disease or comorbidity,^28 33^ major life-changing event or crisis^28^,^3034^ and COVID-19 symptoms and history of quarantine,^33 37^ were reported as risk factors of depression across the different population groups.

The duration of ART,^16^ viral load,^19^ CD4 count,^20 21^ HIV symptoms^16 18^ and history of depression^18^ was significant risk factors for depression among people living with HIV. Burn-out was a common risk factor for depression among students and nurses.^28 41^ Lack of satisfaction in current relationships,^36^ conflict with a partner^36^ and domestic violence^35^ were risk factors for depression among pregnant and lactating mothers. Difficulty feeding the baby and problems with the baby’s sleep were risk factors for postpartum depression.^36^ Furthermore, unplanned pregnancy, abortion and being single were significant predictors of depression among teenage mothers.^35^

### Anxiety

Refugee status, age and female gender were risk factors for anxiety among children and adolescents in the Far North region.^39^ On the other hand, the male gender, in addition to COVID-19 complications and episodes of depression, was shown to be risk factors for anxiety among adult COVID-19 patients.^33^

### Risk factors for other common MHDs

A history of anxiety disorder, depression and a family history of ADHD were all risk factors for ADHD.^27^ Females were at increased risk for mood disorders^27 39^ compared with males while the reverse was true for alcohol use.^26^ Age was shown to be a significant predictor of PTSD.^39^

### Management

Two included studies focused on the management of depression among people living with HIV as a strategy to improve adherence to ART and reduce viral load (online supplemental table 5).^23 24^ Gaynes *et al*^23^ employed the measurement-based care (MBC) approach to the management of depression among people living with HIV in the Bamenda, Northwest Region of the country. The MBC is an evidence-based approach to depression that provides best-practice care and medication with the help of a non-physician, called a depression care manager (DCM). The DCM guides decision-making regarding the time of initiation of antidepressants, dosage and duration of treatment. Implementing this strategy resulted in an average reduction in CD4 count of 16, a 10% increase in the number of individuals reporting adherence of 95% or higher and a significant reduction in depression indicated by the PHQ score −12.8 (−14.2 to –11.3).^23^ Ndenkeh *et al* implemented a psychoeducational intervention that relied on educating participants about the condition and its treatment, coupled with emotional support to increase treatment adherence. By implementing this management approach, the prevalence of moderate to severe depression changed from 9% at baseline to 0.8% at 12 months while adherence changed from 88.7% at baseline to 100% at 6 and 12 months.^24^

## Discussion

To the best of our knowledge, this study is the first to synthesise evidence on MHDs in Cameroon, and we hope it will serve as a baseline for future reviews as it describes the distributions of MHDs, as well as their associated risk factors. The main findings of this review are the high prevalence of common MHDs among different groups, including people living with HIV, children and adolescents, students, postpartum women, and hospital patients with different diseases. Many risk factors were also identified in relation to different mental health conditions and populations. More so, our assessment of findings from two intervention programmes for the management of depression was promising as the findings have significant implications for planning and policy improvement for MHDs in Cameroon.

The high prevalence of common MHDs suggests the need to recognise these disorders as an urgent public health priority. The high prevalence is indicative of a lack of availability and access to adequate care for mental health conditions in Cameroon, coupled with an insufficiency of human resources to sustainably address mental health challenges.^42^ This calls for the institution and careful monitoring of the implementation of mental health programmes as part of the primary healthcare service and ensuring that these services are accessible to all populations, including hard-to-reach communities. Previous studies have demonstrated that improving access and decreasing costs to adequate mental health services are crucial to reducing the high burden of mental health conditions.^43^ Additionally, strategies such as digital mental health (DMH) have been used in other settings to close the treatment gap for MHDs. This strategy entails using digital health technology for diagnosing, treating, preventing and providing support for mental health cases.^44^ The introduction of DMH in Cameroon could yield promising results in closing the treatment gaps and addressing traditional challenges in mental healthcare.^45^ It is essential that policy and implementation efforts reflect the burden of these conditions within the community.

The prevalence estimates varied widely between studies assessing MHDs within a particular population and between populations. For example, the prevalence of depression varied from 3.4 among individuals newly diagnosed with HIV to 63% among persons who have been active on ART. Several reasons could possibly explain the wide range in prevalence. First, this could be differences in the characteristics of the sample in the included primary studies. It is worth nothing that the low prevalence was recorded among newly diagnosed individuals who may be ‘relatively healthier’ and free from depression as opposed to the high prevalence recorded among individuals who are active on ART. Living with HIV and the side effects of ART might either increase the severity of depressive symptoms or increase the chances of developing depression. Second, differences in the measurement instrument used in both studies could account for a wide range recorded due to differences in the sensitivity of the instruments, without ignoring the possibility of measurement error. A meta-analysis of studies among the same population that used the same instrument to capture the presence of depression would yield more precise and reliable estimates.

Our review identified several risk factors for MHDs across the different studies. Some risk factors, such as poverty and the presence of other chronic diseases, were identified across several studies and in different populations. Burn-out, on the other hand, is a significant risk factor among nurses and medical students while lack of satisfaction in current relationships among pregnant and lactating women. The presence of HIV symptoms among people living with HIV was a significant risk factor. These results suggest the need for structural and policy changes to identify and address the root causes of such risk factors across all populations, with keen attention on high-risk groups, as reported in this study. In addition, most of the included studies were performed on populations with special interests (eg, HIV, cancer and diabetes), which indicate a lack of studies and/or data on MHDs in the general population especially in rural areas.

The results of our review also highlight the effectiveness of MBC and psychoeducation intervention for the management of depression among people living with HIV. Adapting these interventions in different communities and risk groups could yield promising results for managing mental health conditions. Therefore, implementing such interventions in the primary healthcare setting, targeting high-risk groups or the whole population has huge potential to reduce the burden of MHDs in Cameroon. We believe that the development, adaptation and implementation of such interventions should be coupled with educating healthcare workers and beneficiary populations on the need and conduct of such interventions. Additionally, aspects of information, education and communication on the availability and ways to access the available interventions for the management of MHDs should be brought closer to the communities at large.

The findings of our systematic review are similar to other reviews conducted in SSA, indicating a high prevalence of MHDs^46 47^ and a sparsity of interventions for managing mental health conditions.^48^ It is, however, worthy of mentioning that, though in general, there exists a high burden of MHDs and limited intervention for the management of these disorders in SSA, the individual countries are at different levels in the implementation of country-level strategies towards addressing the burden of MHDs. The results here are, therefore, more reflective of the situation in Cameroon than the SSA region as a whole. In Cameroon, the following steps have been taken in the last decade, including the creation of a harmonised mental health curriculum for medical students in 2015, the inclusion of mental health in the national health development plan in 2016, and ratification of the UN convention on the right of people with disability in 2021.^49^ However, Ngasa *et al* pointed to the need for the development of legislation to guide mental healthcare and indicate the central role of such legislation in providing high-quality mental health services in Cameroon.^49^ In the same light, the WHO’s Comprehensive Mental Health Action Plan 2013–2030 urges member states to take actions such as ensuring the availability of mental health and psychosocial support services in emergency and disaster situations as well as encouraging intersectoral initiatives for the prevention of MHDs over the life course and putting in place approaches to reduce stigma among individuals with MHDs.^50^

### Strengths and limitations

This study is the first in Cameroon to systematically search and synthesise evidence on common MHDs. We extensively searched the literature for the mental health conditions included in this systematic review, synthesised the results, and presented information on the prevalence, risk factors and management of common MHDs. We mapped the state of research on common mental health conditions in Cameroon, indicating where research has been done on common MHDs in Cameroon. Despite our attempt to be more inclusive in our search and screening, there is always a possibility of missing studies when conducting a systematic review. However, we are confident that if a study was missed, it is unlikely that the result of such a study will be different from the results synthesised here. Despite these, our review reveals the huge gap in research on MHDs in Cameroon, given that the majority of studies were performed in five major cities: Yaoundé, Bamenda, Douala, Limbe and Buea with a few other cities having fewer than five studies (online supplemental figure 1). This review highlights the need for studies in other cities that have not yet been studied, and particularly, in rural settings since their prevalence and risk factors might differ from that recorded in the included studies conducted majorly in urban settings.

Also, very few studies were identified for some MHDs. For example, only a single study was included on ADHD among medical students. Such findings might not be representative of that population in Cameroon. Interpretation must be done in consideration of this limitation.

## Conclusion

This review provided an overview of the burden of common MHDs in Cameroon and revealed a high prevalence of MHDs among different groups. Several important risk factors identified in this study, coupled with the high prevalence, point to the need for policies and multisectorial actions geared towards addressing the high burden of MHDs. Our results also highlight two promising interventions for the management of depression that can either be adopted or adapted to different settings and populations. Increased participation and priority on MHDs at all levels, including research where a huge gap remains to be filled, is crucial in reducing the burden of MHDs in Cameroon.

## supplementary material

10.1136/bmjph-2023-000224 online supplemental file 1

## Data Availability

Data sharing not applicable as no datasets generated and/or analysed for this study.
