# Supplementary material for: Prevalence, risk factors and management of common mental health disorders in Cameroon: a systematic review
Source: BMJ Public Health. 2024 May 30;2(1):e000224. doi: 10.1136/bmjph-2023-000224 (PMC11812747; doi:10.1136/bmjph-2023-000224)
Supplement: online supplemental file 1 [file bmjph-2-1-s001.pdf]

Supplementary Table 1: Complete search strategy

1	Mental Health/ or Mental Disorders/ or Anxiety Disorders/ or Depression/ or Disruptive, Impulse Control, and Conduct Disorders"/ or "Attention Deficit and Disruptive Behavior Disorders"/ or Bipolar Disorder/ or Attention Deficit Disorder with Hyperactivity/ or Suicide, Assisted/ or Suicide, Completed/ or Suicide/ or Suicide, Attempted/ or Substance-Related Disorders/ or Self-Injurious Behavior/ or Psychotic Disorders/ or Schizophrenia/ or Stress Disorders, Post-Traumatic/
2	((mental adj4 ("health" or "disorder" or "illness")) or anxious or depress or delir* or psychiatr* or mental illness*.mp).mp.
3	Mental Health Recovery/ or Community Mental Health Services/ or Community Mental Health Centers/ or Mental Health Services/
4	(intervention* or treat* or therap* or program* or service* or prevent*).mp.
5	1 or 2 or 3 or 4
6	Cameroon/ or Cameroun.mp or Cameroon.mp
7	5 AND 6

Supplementary Table 2: Study characteristics

Author Last name	Title	Language	Location	Participants	Age range	Sample size	year of data collection
Massongo et al,2022	Sleep Apnea Syndrome: Prevalence and Comorbidity with Other Non-communicable Diseases and HIV Infection, among Hospitalized Patients in Yaounde, Cameroon	English	Yaoundé	Hospitalised patients	28-87	111	2017
Parcesepe et al, 2021	Gender, Mental Health, and Entry Into Care with Advanced HIV Among People Living with HIV in Cameroon Under a National 'Treat All' Policy	English	Limbe, Bamenda, Yaounde	People living with HIV iniating ART at 3 Hospital	≥21	426	2017
Parcesepe et al, 2022	Depressive Symptoms, Gender, Disclosure, and HIV Care Stage Among People Living with HIV in Cameroon	English	Limbe, Bamenda, Yaounde	People living with HIV	≥18	12,507	2016-2020

Ebob-Anya et al,2022	Psychosocial distress and the quality of life of cancer patients in two health facilities in Cameroon	English	Douala	Cancer patients	18-73	120	2020
Nicolet et al,2021	Teenage Mothers in Yaounde, Cameroon,Risk Factors and Prevalence of Perinatal Depression Symptoms	English	Yaoundé	Teenage mothers	20 or less	1307	2014-2017
Ngasa et al,2021	Prevalence and factors associated with anxiety and depression amongst hospitalised COVID-19 patients in Laquintinie Hospital Douala, Cameroon	English	Douala	Covid-19 patients at Laquintinie Hospital		285	2021
Njim et al, 2020	Determinants of depression among nursing students in Cameroon: a cross-sectional analysis	English	Bamenda; Buea	Nursing students	22.28(mean age)	447	2018
Djatche et al, 2022	A cross-sectional analysis of mental health disorders in a mental health services-seeking population of children, adolescents, and young adults in the context of ongoing violence and displacement in northern Cameroon.	English	Kolofata, Makary, Mokolo, Mora, and Mozogo	among children, adolescents, and young adults living Far North region		948	2018-2019
Mbanga et al,2019	Burnout as a predictor of depression: a cross-sectional study of the sociodemographic and clinical predictors of depression amongst nurses in Cameroon.	English	Bamenda; Buea; Limbe, Mutengene	Nurses	20-55	143	jan-Jun 2018

Parcesepe et al, 2021b	Mental health and initiation of antiretroviral treatment at enrolment into HIV care in Cameroon under a national "treat all"� policy: a cross-sectional analysis	English	Limbe, Bamenda, Yaounde	People with HIV initiating ART	≥21	420	2019-2020
Nguendo-Yongsi et al,2022	Access to health care in African cities: therapeutic pathways of city dwellers with mental health problems in Yaounde Cameroon	English	Yaoundé	Community inhabitants	≥15	944	2015-2017
Ngasa et al,2017	Prevalence and factors associated with depression among medical students in Cameroon: a cross-sectional study.	English	Buea, Bamenda,Yaoundé, Douala	Medical students	18-28	618	2015-2016
Fodjo et al, 2021	Fear and depression during the COVID-19 outbreak in Cameroon: a nation-wide observational study.	English	Nationwide	Everyone (online survey)	≥18	7381	2020

Njim et al, 2019	Burnout as a correlate of depression among medical students in Cameroon: a cross-sectional study.	English	Bamenda, Douala, Buea	Medical students		413	2018
Njuwa et al ,2020	Factors associated with symptoms of attention deficit hyperactivity disorder among medical students in Cameroon: a web-based cross-sectional study.	English	Buea, Bamenda,Yaoundé, Douala, Banganté, Kumbo	Medical students	18-32	491	2018
Abelson et al, 2019	Lifetime experiences of gender-based violence, depression and condom use among female sex workers in Cameroon.	English	Yaoundé, Douala, Bertoua, Kribi and Bamenda	Female sex workers and men having sex with men	≥18	2165	
Kouotou et al,2016	Acne in Cameroon: Quality of life and psychiatric comorbidities	French	Yaounde	Patients diagnosed with acne after consultation with a dermatologist	Between 13 and 56 years	181	2015
Parcesepe et al,2021C	Common mental disorders and intimate partner violence against pregnant women living with HIV in Cameroon: a cross-sectional analysis.	English	Yaoundé	Pregnant women(in 3rd trimester) living with HIV	≥18	230	

Asangbeh et al,2016	Predictors of depression among patients on art in a rural health district in North West Cameroon.	English	Yaoundé	People living with HIV/AIDS(PLWHA)	≥21	202	2012
Kanmogne et al,2017	Depressive symptoms in HIV-infected and seronegative control subjects in Cameroon: Effect of age, education and gender.	english	Yaoundé	People living with HIV		290	2008-2015
Kehbila et al, 2016	Prevalence and correlates of depressive symptoms in adult patients with pulmonary tuberculosis in the Southwest Region of Cameroon.	English	Limbe, Kumba	People diagnosed with TB	≥21	265	2015
Gaynes et al,2012	Prevalence and predictors of major depression in HIV-infected patients on antiretroviral therapy in Bamenda, a semi-urban center in Cameroon.	English	Bamenda	People living with HIV		400	2010
Voundi et al,2022	Association between depression and viral load in people on antiretroviral treatment followed at the Yaounde Central Hospital in Cameroon.	French	Yaoundé	People living with HIV	46 (mean)	205	November 2019 - July 2020
L'akoa et al,2013	Prevalence and correlates of depressive symptoms in HIV-positive patients: a cross-sectional study among newly diagnosed patients in Yaounde, Cameroon.	English	Yaoundé	Newly diagnosed HIV patients	≥18	100	2011

Metuge,2022	Prevalence and factors associated with substance use among students in tertiary institutions in Buea, Cameroon.	English	Yaoundé	Students	≥18	650	2019
Adama et al,2015	Prevalence and Risk Factors of Postpartum Depression in Yaounde, Cameroon	English	Yaounde	Post patum women	15-45	214	2013-2014
Ngum et al, 2017	Depression Among HIV/AIDS Patients on Highly Active Antiretroviral Therapy in the Southwest Regional Hospitals of Cameroon: A Cross-Sectional Study	Eglish	Buea, Limbe	People living with HIV	≥21	300	2014-2015
Aroke et al,2020	Prevalence and factors associated with depression among type 2 diabetes patients in a Reference Hospital in Cameroon	English	Douala	Diabetes patients	≥21	177	December-2014-March2015
Mboua et al, 2021	Impact of the resumption of classes on the mental health of students of the Faculty of Letters and Social Sciences of the University of Dschang, in the context of Covid 19	English	Dschang	students	≥15	254	
Wamia et al, 2019	The Role of Support Groups in the Management of Depression amongst People Living with HIV at Regional Hospital of Bamenda	English	Bamenda	People living with HIV		152	

Gaynes et al,2015	Changes in HIV Outcomes Following Depression Care in a Resource-Limited Setting: Results from a Pilot Study in Bamenda, Cameroon	English	Bamenda	Depressed people on ART		55	2011
Ndenkeh et al, 2022	Depression management and antiretroviral treatment outcome among people living with HIV in Northwest and East regions of Cameroon	English	santa,(bamenda); Abong bang (east)	People living with HIV on ART		262	2019-2021

Supplementary Table 3: Prevalence of common mental health disorders.

Author, year	Depression n(%)	Anxiety	Post-traumatic disorder	Mood disorder	Alcohol use	Schizophrenia	Bipolar disorder	ADHD
**Massongo et al, 2022**	[a]Moderate to severe 21(20.8)	[b]Anxiety 1(1)						
**Parcesepe et al, 2021**	[a]Moderate to severe 86 (20.5)	[b]Moderate to severe 51(12.3)	[c]Probable 64(15.5)					
**Parcesepe et al, 2022**	[d]Depression 1570(12.5)							

**Ebob-Anya et al, 2022**	[e]Moderate to Severe 56 (47.5)	[e]Moderate to severe 59 (50)						
**Nicolet et al., 2021**	[f]Depression 915 (70)							
**Ngasa et al, 2021**	[e]Abnormal 172(60.35) Borderline 105 (36.84)	[e]Abnormal 232 (81.4) Borderline 51 (17.89)						
**Njim et al., 2020**	[a]Moderate to severe 118 (26.4)							
**Djatche et al., 2022**		[c]Anxiety 230 (24.7)	[c]PTSD 161(17.3)	[c]Mood disorder 76(8.2)				
**Mbanga et al., 2019**	[a]Moderate to severe 43 (31.04)							

**Parcesepe et al., 2021**	[a]Moderate to severe 83 (20.0)	[b]Moderate to severe 49 (12.0)	[c]Probable 63 (15.5)		[g]Alcohol use 56 (13.4)				
**Nguendo-Yongsi et al, 2022**	[h]Prevalence of depression in Male: 9.3% female :18.5%	[h]Prevalence of anxiety in Male: 10.8% Female: 13.1%			[h]Male:12.5 ; female :10.5	[h]Male :10.1 ; female 7.9%	[h]Male : 8.6% ; Female :13.6		
**Ngasa et al, 2017**	[a]Moderate to severe 189 (30.6%)								
**Fodjo et al, 2021**	[a]Moderate to severe 617 (8.4)								
**Njim et al, 2019**	[a]Moderate to severe 95 (23)								
**Njuwa et al , 2020**	[I] History of Severe Depression 59(12%)	[I] History of Anxiety disorder 153(31.2)							[J]ADHD 120(24.4)

**Abelson et al, 2019**	[a]Depression 1067 (49%)								
**Kouotou et al, 2016**	[a]Depression 11 (6.1)	[b]Anxiety 14(7.7)							
**Parcesepe et al, 2021C**	[K]Probable common mental health disorder 97(42.2) for ⅞ cut off	[K]Probable common mental health disorder 97(42.2) for ⅞ cut off							
**Asangbeh et al, 2016**	[a]Depression 48 (28.7)								
**Kanmogne et al, 2017**	[l]Moderate to severe 47 (33.73)								
**Kehbila et al, 2016**	[a]Moderate 65(24.5)								

**Gaynes et al, 2012**	[mn]Depression in the last year 29 (7.25)							
**Voundi et al, 2022**	[e]Depression 10 (4.8)							
**L'akoa et al, 2013**	[a]Moderate to severe 63%				[o]Alcohol use 17%			
**Metuge, 2022**					[p]Alcohol use 553 (98.5)			
**Adama et al, 2015**	[f]Depression 50 (23.4)							
**Ngum et al, 2017**	[a]Moderate to severe 10 (3.4)							
**Aroke et al,2020**	[a]Moderate to severe 29.4%							
**Mboua et al, 2021**	[q]Depression 80 (31.5)	[q]Anxiety 75 (29.5)						

**Wamia et al, 2019**	[a]Moderate to severe 32 (21.05)							

[a]Patient health questionnaire-9(PHQ-9); [b]General Anxiety Disorder-7(GAD-7)scale; [c]Diagnostic and Statistical Manual of Mental Disorders 5th edition(DSM-5); [d]Patient Health questionnaire-2(PHQ-2); [e]Hospital Anxiety and Depression scale(HADS); [f]Edinburgh Postpartum Depression Scale(EPDS); [g]Alcohol Use Disorders Identification Test(AUDIT), [h]Mini-International Neuropsychiatric Interview; [i]Past history of physician/specialist confirmed diagnosis; [j]ADHD Self-Report Scale (ASRS) v1.1 screener; [k]WHO Self-Reporting Questionnaire(SRQ-20); [l]Beck Depression Inventory-II(BDI-II); [m]Quick Inventory of Depressive Symptoms(QIDS) & [n]The Composite International Diagnostic Instrument(CIDI); [o]Cutting down, Annoyance by criticism, Guilty feelings, Eyeopeners questionnaire(CAGE); [p]Global Assessment Program on Drug Abuse (GAP-Toolkit Model 3); [q]Depression Anxiety and Stress Scale(EDAS).

Supplementary Table 4: Risk factors for common mental health conditions.

First author	Participants	Sample size	Mental health disorder	Instrument	Measure of association	Insignificant factors	Significant factors
Nicolet et al,2021	Teenage mothers	1307	Perinatal depression	The Edinburgh Postnatal Depression Scale (EPDS)	Odds Ratio (adjusted)	More than 3 kids 0.95(0.68–1.37); Poverty 0.79(0.61–1.03); refugee status 0.50(0.27–1.07); living in disaster or conflict zone 0.45(0.23–1.11); Inability to confide in a partner 0.98(0.74–1.34); polygamous family 0.77(0.51–1.20);	Single or separated 1.34(1.12–1.60); Unplanned pregnancy 1.33(1.14–1.56); depression before birth 1.50(1.02–2.27); Abortion 2.60(1.03–7.14); Domestic violence 1.76(1.12–2.83)
Ngasa et al,2021	Covid-19 patients at Laquintinie Hospital	285	Depression	HADS	odds ratio(adjusted)	Gender (aOR:1.0); Employment status(aOR:1.20); alcohol consumption (aOR:1.01);	age > 35 years (aOR:2.03); presence of comorbidity (aOR:1.68); Obesity (aOR: 1.78); hypoxaemia (aOR:2.14); presence of COVID-19 complications (aOR: 1.28) and anxiety (aOR: 4.6)
			Axiety	HADS	Odds Ratio (adjusted)	Marital status(aOR:1.43); Age group (aOR:1.48); Education (aOR:1.16); Employment status(aOR:1.30); History of depression(aOR: 1.49)	Male gender (aOR: 1.89); Hypoxaemia (aOR: 2.20); Presence of COVID-19 complications (aOR: 1.61) and current episode of depression (aOR: 4.14,)

Njim et al, 2020	Nursing students	447	Depression	PHQ-9	Odds Ratio (adjusted)	marital status (aOR: 0.95); personal relationship(aOR:1.64); Income(aOR:0.64); presence of chronic disease (aOR:1.68); Age(aOR:0.95); Number of children(aOR:0.96)	Burnout score(aOR:1.18); Level of studies(aOR:0.72); life-changing crisis(aOR:2.13)
Djatche et al, 2022	among children, adolescents, and young adults living Far North region	948	Axiety	DSM-5	Odds Ratio (adjusted)		Status [Refugee 0.30(0.14–0.67) , IDPs 0.54 (0.36–0.80) compared to host population] ; Age[2.42 (95% CI 1.30, 4.48) for 15-years olds, 3.26 (95% CI 1.79, 5.95) for 20-24 years old compared to 10-14 years] ; Gender[1.71(1.17–2.50) females]
			mood disorder	DSM-5	Odds Ratio (adjusted)	Status, Age	Gender 2.18(1.16–4.10) females compared to males
			Trauma and stress-related disorder	DSM-5	Odds Ratio (adjusted)	Gender , Status	Age-group 20-24 1.79(1.02–3.13) compared to 10-14
Mbanga et al,2019	Nurses	143	Depression	PHQ-9	odds ratio (adjusted)	Majority of shifts 2.15(0.48, 9.66); Age 0.94(0.86, 1.03)	Number of night shifts a week 1.58(1.01, 2.48); burnout score 1,21 (1.08, 1.35)

Ngasa et al,2017	Medical students	340	Depression	PHQ-9	odds ratio(adjusted)	Age(years)0.69(0.45–1.06); Regret studying medicine 1.64(0.96–2.78); Alcohol consumption 1.12(0.78–1.59)	Resit 2.06(1.35–3.16); Chronic diseases 3.70(1.72–7.94); Major life event 2.17(1.32–3.58); Level of study :clinicals 4.26(2.71–6.71); Gender :female 1.59(1.06–2.37)
Fodjo et al, 2021	Everyone (online survey)	7381	Depression	PHQ-9	Odds ratio (adjusted)	Age(in years) 0.994(0.986–1.003); Socioeconomic status; Profession; Living alone in household 1.098(0.986–1.224); History of violence/discrimination 0.949(0.816–1.104); student/healthcare worker 1.095(0.958–1.252)	COVID-19 information from social media 0.831(0.758–0.912);COVID-19 information from social media 0.831(0.758–0.912); Flu-like symptoms during the past 14days 2.296(2.050–2.570); Fear of COVID-19 1.114(1.102–1.127); History of quarantine/isolation for COVID-19 1.568(1.422–1.728)

Njim et al, 2019	Medical students	413	Depression	PHQ-9	Beta-Coefficient	Number of hours studying −0.01(−0.32 to 0.30); Gender 0.14(−0.99 to 1.28); Personal relationship-(Yes) −0.91(−2.18 to 0.36); Sufficient monthly income(yes) −0.28(−1.45 to 0.89); Regret choice of medical studies 0.09(−2.16 to 2.35); Alcohol consumption 0.54(−0.71 to 1.79); Recreational drug use 2.21(−1.10 to 5.52); Satisfaction with results −0.94(−2.12 to 0.32)	Presence of chronic illness 3.19(0.96 to 5.42); Life changing crises in past 6 months 1.29(0.13 to 2.45); Number of children −2.26(−3.70 to -0.81); burnout score 0.32(0.22 to 0.42)
Njuwa et al ,2020	Medical students	491	ADHD	ADHD Self-Report Scale (ASRS) v1.1 screener	odds ratio (adjusted)	Age (in years) 1.01 (0.92 to 1.10); Gender 0.83 (0.52 to 1.33);	History of anxiety disorder (yes) 2.06(1.25 to 3.36); History of severe depression (yes) 3.49 (1.82 to 6.77); History of chronic diseases (yes/ref: no) 2.96 (1.49 to 5.86); Family history of ADHD (yes/ref: no) 3.38 (1.04 to 10.44)
Asangbeh et al,2016	People living with HIV/AIDS(PLWHA)	202	depression	PHQ-9	odds ratio(adjusted)	age; Sex 0.85(0.35–2.06); marital status (singled/widowed) 0.77(0.37–1.60); level of education(primary/less) 0.53(0.24–1.19);	Income 1(1.18–5.18); CD4 count; Duration on ART(≥2years) :1(0.31–0.97); Presence of HIV/AIDS symptoms(no) :1(2.09–8.81)

Kehbila et al, 2016	People diagnosed with TB	265	depression	PHQ-9	odd ratio(adjusted)	Age>40years :1.3(0.7–2.4); BMI [<18.5kg/m2: 0.3(0.1–1.3), 18.5–24.9kg/m: 0.4(0.1–1.4)]; Treatment phase-intensive :1.2(0.6–2.1); Patient status-Hospitalized 1.2(0.6–2.3); positive sputum smear: 1.9(0.9–3.8);	Sex-female :3.0(1.7–5.5); Co-morbidity-yes:2.5(1.2–6.5); Family history of mental ilness-yes:2.5(1.3–5.4); Discontinuation of treatment-yes: 8.2(1.1–23.3); treatment status-retreatment : 11.2(5.2–31.1)
Gaynes et al,2012	People living with HIV	400	Depression	Quick Inventory of Depressive Symptoms embedded (QIDS) in the Composite International Diagnostic Instrument (CIDI)	odds ratio(adjusted)	Sex-female: 0.61(0.23,1.64); Age :1.03(0.61,1.73); Marital status [previously married 1.57(0.53,4.62), never married :1.24(0.36,4.32)] ; Education-greater than primary school 1.03(0.38,2.81); Rural residence 0.72(0.29,1.81)	HIV symptoms 1.22(1.05,1.42) ; Number of prior lifetime depressive episodes[One 5.42(1.65,17.75), Two/more 11.38(4.04,32.04)]
L'akoa et al,2013	Newly diagnosed HIV patients	100	Depression	PHQ-9	Odds ration(adjusted)	Age1.03(p-value-0.28); Gender(male)2.42(p-value:0.23); Marital status(couple)1.17(p-value:0.78); Educational level(university)2.40(p-value:0.24); Area of residence(urban)2.25(p-value:0.25); Area of residence(urban)2.25(p-value:0.25); Personal past history of depression(no)0.55(p-value:0.59); Antiretroviral	Alcohol abuse(no)44.66(p-value:0.003); CD4cellcount*3.30(p-value:<0.0001)

						treatment(yes)1.95(p-value:0.28)	
Metuge,2022	Students	650	Alcohol use	Global Assessment Program on Drug Abuse (GAP) (Toolkit Model 3)	Odds ratio(adjusted)	Age-15 - 25 years : 1.406 (0.931-2.122); Religion-Christian: 0.799 (0.238-2.682); Living condition-with parents/Guardian :0.716 (0.518-0.991); Year of study -1-2 years: 0.730 (0.528-1.011)	Median monthly income (≤27000): 0.682 (0.490-0.950); Sex-male : 1.917 (1.337-2.748);
Adama et al,2015	Post patum women	214	Depression	Edimburg Postpartum Depression Scale (EPDS)	Odds ratio(adjusted)	Loss of a job recently:1.93 (0.64 - 5.78); Abortion history :2.73 (0.95 - 7.81); Unplanned pregnancy :1.08 (0.27 - 4.35); Unwanted pregnancy :1.64 (0.66 - 4.12); Depressive symptoms during pregnancy :1.66 (0.71 - 3.89); Anxiety during pregnancy : 1.36 (0.56 - 3.31); Lack of emotional family support:2.24 (0.79 - 6.35); Can trust the father of the child:1.18 (0.48 - 2.91); Cannot rely on the father of the child: 3.10 (0.76 - 12.64); Lack of adequate material support the father of the child:1.28 (0.30 - 5.43); Lack of adequate emotional support of the child's father 1.32 (0.45 - 3.93)	lack of satisfaction in the marital relationship: 6.91 (3.29 - 14.49); Recent conflicts with the partner : 2.55 (1.05 - 6.18); Recent financial problems: 3.85 (1.44 - 10.28) ; Baby blues: 3.52 (1.48 - 8.41); Difficulties in feeding the baby: 3.51 (1.26 - 9.82) ; Problems with the baby's sleep : 2.02 (1.06 - 3.81)

Ngum et al, 2017	People living with HIV	300	Depression	PHQ-9	Odds ratio(adjusted)	Marital status-unmarried 1.23 (0.68–2.25);Living companion-No 1.72 (0.86–3.45)	Age≤40: 2.13(1.20–3.70); CD4 count≤200: 3.70(1.45–9.09); Employment status-unemployed 2.38 (1.26–4.50);
Voundi et al,2022	People living with HIV	205	Depression	HAD	odds ratio(adjusted)	Religion: christianity 0,335 (0,07-1,58)	Elevated viral Load 14,24 (3,61-56,14)
Aroke et al,2020	Diabetes patients	177	Depression	PHQ-9	Odds ratio(adjusted)	Age (≤48 vs >48 years): 2.39 (0.60–9.48); Unemployed (yes vs no): 0.86 (0.37–2.00); Physical inactivity (yes vs no):1.61 (0.72–3.57); Nephropathy (yes vs no):0.42 (0.10–1.72); Insulin use (yes vs no) :1.44 (0.57–3.64); cerebrovascular accident: 1.71 (0.27–10.77)	Major life event (yes vs no):8.38 (2.79–25.15); Neuropathy (yes vs no): 3.25 (1.47–7.19)

Supplementary Table 5: Management of common mental health conditions

author Last name	Location	Outcome	Intervention	Pre-intervention	Post-intervention
Gaynes et al, 2015	Bamenda	Depression score; CD4 count; adherence	Measurement-Based Care (MBC) is an evidence-based depression management strategy that relies on the delivery of best-practice care and medication for the management of depression by psychiatric and non-psychiatric medical practitioners. A non-physician Depression care Manager (DCM) who could be a nurse, social worker etc. Provides support in the decision making regarding the initiation, dosage and duration of antidepressant treatment. Regular measurement of depressive symptoms and side effects is an integral part of the MBC strategy. This strategy is particularly useful in low resource setting where the is shortage of trained medical personnel such as physicians and psychiatrist	Mean (range) PHQ-9 score: 14.4(13.1, 15.6). Baseline CD4 count 436(2, 860); 53% of participant reported adherence of 95%. Or higher	CD4 count improved: mean difference (96% CI): 16(-47, 79); participant-reporting adherence of 95%. Or higher increased by 10%(95%CI:-11, 32); significant improvement in PHQ score(mean difference -12.8(-14.2, -11.3)

Ndenkeh etal, 2022	santa,(Bamenda) ; Abong bang (East)	ART adherence and Viral load suppression	Psycho-education intervention: This is an education-oriented strategy for depression management and relies on the systematic transfer of knowledge about an illness and its treatment to the patient in a bid to increase treatment adherence and efficacy. This intervention also incorporates emotional support and motivational aspects to help patients cope. The intervention also included pharmacotherapy when needed.	42(11.4%) of participants had moderate to severe depression. Particularly 31(8.4%) moderate depression while 10(2.7%) and 1(0.3%) had moderately severe and severe depression respectively. No difference observed between intervention and control group at baseline. Moderate to high adherence was reported by 158(88.7%) in the intervention group and 171(89%) in the control group at baseline.	**Control group**: The prevalence of moderate to severe depression changed from 26(13.5%), 27(16.8) and 7(5.1%) at baseline, 6 months and 12 months**. Intervention group**: The prevalence of moderate to severe depression changes from 16(9%) to 2(1.6%) and 1(0.8%) at baseline, 6months and at 12 months. Moderate to High adherence improved from 171(88.7%) to 124(100%) at 6 and 12 months while in the control group, moderate to high adherence changed from 171(89%) at baseline to 157(97.5%) and 135(97.8%) at 6 months and 12 months respectively. No significant difference in viral load was observed. However is important to note that not all results regarding participants viral load were ready at the end of the study

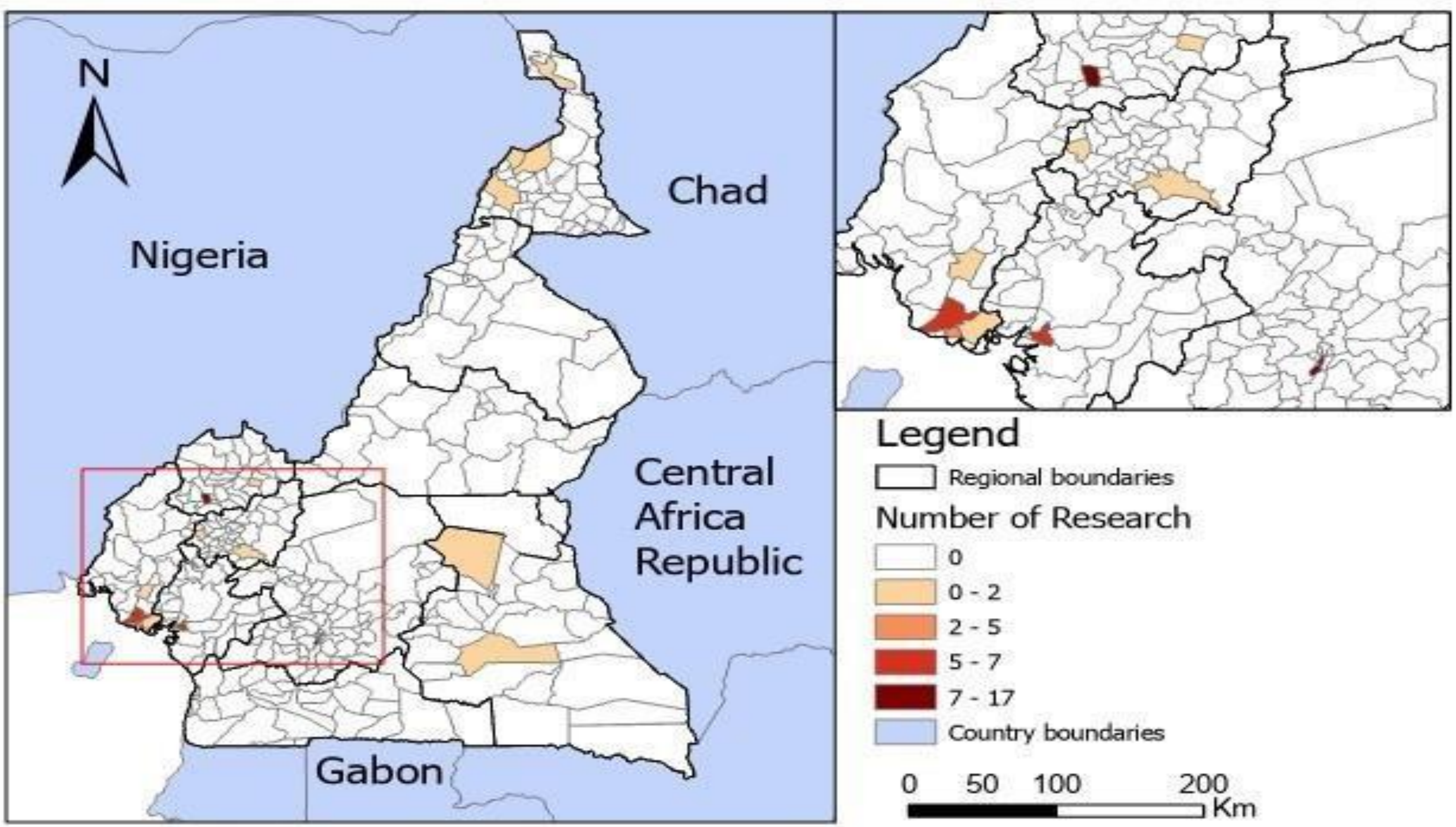

Location of data collection for all included studies in the systematic review.
This map provides a pictorial view of the location where data was collected for all the studies, and mental health conditions included in this systematic review. The map provides a starting point on which further improvement can be based for visually depicting areas where research is lacking. This map should be interpreted considering the inherent limitations of systematic reviews, which is the possibility that some studies were missed.

Supplementary Figure1: A map showing the location of data collection for all included studies.
